# Planning and Reporting of the Histomorphometry Used to Assess the Intestinal Health in Fish Nutrition Research—Suggestions to Increase Comparability of the Studies

**DOI:** 10.3389/fvets.2021.666044

**Published:** 2021-04-21

**Authors:** Ioannis N. Vatsos

**Affiliations:** Faculty of Biosciences and Aquaculture, Nord University, Bodø, Norway

**Keywords:** fish, nutrition, semiquantitative, quantitative, histology, histomorphology

## Introduction

The intestine of all animals can exhibit dramatic and rapid functional and structural alterations in response to different dietary factors. Therefore, histomorphometry can assess, in a quantitative or semi-quantitative manner, the overall effect of any feed ingredient on the microscopic structure of the organ, providing, at the same time, a good indication on its effects on the intestine functions.

Like any other method, intestine histomorphometry has many steps, starting from the collection of appropriate and sufficient tissue samples, to the statistical analysis of the measurements. The aim of the present opinion paper is not to provide a detailed discussion on all steps included, but to provide suggestions on the critical points regarding the planning and reporting of the histomorphometric assessment that is routinely used in fish nutrition research. Based on these suggestions and following the ARRIVE recommendations, Table 1, presents a checklist for the authors to consider, in order to increase the comparability and ultimately the repeatability of the studies.

**Table 1:** Checklist for the planning and reporting of intestine histomorphometry in fish nutrition research.

**Point**	**Recommendation**
Scope	Define clearly the scope of histomorphology and how this assessment is related to any other studies included in the article.
Type of assessment	Explain why a semiquantitative or a quantitative assessment was selected.
Number of animals sampled per treatment	Justify the number of animals sampled, including an a priori power analysis and an indication of the effect size.
Number of tissue sections and observation areas per section	State and justify the number of tissue sections per fish and per intestine segment used, particularly in relation to the expected effect on the intestine (i.e., localized or more extensive). Within each section, how many observation areas were used for the assessment of the indices and why.
Selection of morphometric indices	Justify the relevance of the selected indices in relation to the overall scope of the article. State any threshold scores/values that are considered meaningful in relation to other results.
Selection of scores	If a semiquantitative assessment is chosen, use a scale of 4–5 scores per index, with clear and complete descriptions.
Statistical methods	Use appropriate statistical methods depending on the nature of the assessment: quantitative or semiquantitative. Carry out correlational tests between this assessment and other obtained results, e.g., growth performance, immune function, gene expression, etc.
Interpretation/implications	Discuss the results in relation to the initial hypothesis and in relation to the other results.

## Collection of Appropriate Tissue Samples—When, Where, How

Generally speaking, intestine tissue samples are collected at the beginning of the trial and later, at different time points. One important piece of information that is rarely mentioned is how the number of sampled animals per group was decided. This is stressed in the ARRIVE guidelines (1), is included in many relevant legislations and is even part of the application to receive approval for the use of the animals in research. As this number is also connected to the observed differences (or lack of) between the treatments, a power analysis should always be included in the methodology and the results. This analysis will also indicate what differences are considered meaningful by the authors (e.g., what the effect size is).

Another piece of information that is also rarely mentioned is the collection of the tissue samples in relation to the last feeding. As even a short-term starvation of 24 h can have an effect on the microscopic structure of intestine (2), this period should always be reported and standardized, if possible.

One thing that often many authors seem to disagree on, is the determination of the different intestine segments. When observing the intestine of fish, in most cases, the organ is not easily divided morphologically into distinct segments, as the intestine of mammals. However, from the anterior intestine to the posterior end, different functions take place and microscopically, at least three different segments can be distinguished, anterior, mid, and posterior. For example, major differences in the microscopic structure, related to the function, include differences in the height of intestinal villi (reduced toward the posterior intestine) and differences in the number of goblet cells (increased toward the posterior intestine). The determination of the proportion of the anterior, mid, and posterior intestine is mostly related to the fish species. However, even for the same species, different authors use different ways to distinguish the different segments, sometimes without even stating the criteria used for this definition. For example, what is indicated as posterior intestine in zebrafish (*Danio rerio*) by Wallace et al. (3) is considerably shorter compared to what is indicated by Wang et al. (4). In trout, anterior intestine is usually considered the segment with the pyloric caeca (5), but sometimes, it can also be the area behind this (6). As the length of the intestine can also be corelated to the overall size of the individual fish, it is therefore preferable if the segments are presented as percentage of the entire length, based again on the actual function of the segment. Adjustments, based on the scope of the study, should be clearly justified in the methodology section.

## Preparation of Tissue Sections for the Evaluation

For any histomorphometric evaluation, an unbiased selection of a sufficient number of observation areas, containing appropriate intestinal folds, is important. Different authors use either longitudinal, or cross sections of the different intestine segments. Although there is limited published information in fish, it appears that longitudinal sections of closed intestine are more appropriate, as Gava et al. (7) concluded for the morphometric analysis of intestine in broilers. Cross sections can also be used, but then, more sections are needed to provide a statistically representative picture of the whole length of the segment, particularly in relation to the developed condition (i.e., is this a multifocal or a diffuse enteritis, extending to the entire segment?). In any case, the criteria for the selection of the number of tissue sections and the observation areas within the sections should be clearly mentioned in the methodology, particularly in relation to the expected variation along the intestine segment.

## Planning a Semiquantitative or a Quantitative Assessment

Semiquantitative histomorphometric evaluation is an easy way to perform a quick screening of many tissue samples, particularly in pilot studies, but it relies heavily on the training/experience of the observer and it has to meet certain criteria (8). On the other hand, quantitative histomorphometry provides with a clear set of measurements or enumerations, requiring little experience by the observer. The key point here is that the measurements should be representative of the entire intestine segment as mentioned earlier. For instance, in one of the most fundamental published morphometric assessment of the soybean meal induced enteritis in salmon (9), it was not mentioned whether the enteritis affected evenly the entire posterior intestine and thus how representative were the results provided by the use of 3–4 cross sections per fish. For both the semiquantitative and quantitative assessment, after the cutting and the appropriate staining, the tissue sections are observed using light microscopy. Photographs of the appropriate observation areas are taken and specialized image analysis software is used for the assessment. For the semiquantitative assessment, for each descriptive index selected, an arbitrary scoring system is suggested (e.g., from 1 to 5), whereas for the quantitative assessment measurement/enumeration of certain indices is carried out. In both assessments, a number of observation areas are arbitrary chosen and depending on the type of data, the mean or the median of all these areas is reported per animal.

The selection of the indices in the different studies, and particularly for the semiquantitative approach, the description and the range of the scoring system, are probably the issues that exhibit the highest variation among the different studies, making them the most critical factors that reduce the comparability between studies. Using soy-related research as an example, the following studies that employed semiquantitative intestine histomorphometry can be used to demonstrate this variation. (a) Kokou et al. (10) used 9 fish per treatment, 5 indices and 3 scores per index, (b) Liu et al. (11), used 6 fish per treatment, 3 indices and 5 scores per index, (c) Venold et al. (12) used 12 fish per treatment, 8 indices and 10 scores per index. Although all three studies provided interesting results and succeeded in identifying some differences between treatments, they did not justify the number of fish used, or why they chose the specific indices and scoring system, so that their results can be easily compared with future studies. Regarding the scoring system, Ward and Thoolen (13) suggested that the indices used should be definable, reproducible, and should have a relevant to the studied subject value. The range of scores should preferably be between 4 and 5, to maintain a reasonable level of sensitivity, maintaining at the same time good repeatability. Common descriptions that often appear in publications, like “normal size,” “increased size,” and “large size,” should be avoided. Any modifications should be justified, as they can affect the comparability between studies.

## Selection of Appropriate Histomorphometric Indices

The most common histomorphometric indices used in fish nutrition studies are: (1) morphology and height of intestinal villi, (2) height of enterocytes, (3) number and size of goblet cells, (4) lamina propria and submucosa thickness, (5) tunica muscularis thickness, (6) supranuclear vacuoles in enterocytes, (7) identification of various inflammatory cells (e.g., lymphocytes) in the epithelium, or in the lamina propria and submucosa (often using immunohistochemistry). Other indices that have been used by some authors are: fractal dimension analysis (to study the architectural complexity of the intestinal mucosa) and microvilli morphology and density. Within the context of fish nutrition, the use of the before mentioned indices, aims to assess the effects of any experimental diets on overall health status of the intestine and the presence of an inflammation. Furthermore, this assessment will support other observations, like growth performance, and indirectly indicate the capacity of the intestine to respond to other factors that might appear, such as various pathogens. However, there are some considerations regarding their use.

### How Relevant Are the Indices and When Do the Values/Scores Become Meaningful?

In mammals, there are some common findings observed in most types of enteritis, like increased infiltration of the intestinal wall by a variety of inflammatory cells (initially in the mucosa and then submucosa), epithelial cell proliferation, changes in the number and size of goblet cells and changes in the villus morphology (14). Consequently, most of the previously listed indices for fish are considered relevant, as the numerous publications on the enteritis induced by plant feed ingredients also demonstrate. However, what is not that clearly demonstrated in fish is when the observations become meaningful and not only statistically different. In fish, for instance, it appears that depending on the experimental design, growth and morphometric indices are not always correlated, as for example, Egerton et al. (15) and Najafabad et al. (16) observed. It should be noted though that in both studies, the indices were assessed separately and this could have potentially reduced the strength of the overall assessment of the organ, as it is discussed below. What is also critical for future studies, is that the studies should include a correlational assessment, to demonstrate how relevant the indices are to the scope of the study and ideally, to set thresholds, above which values/score become meaningful, as for example this is very common in human medicine (17).

### Can the Indices Be Used Separately or a Sum/Average Score Is More Relevant?

For both ways of assessment, the values of the indices are presented either as separate scores, or as a sum/average of the individual indices. The first approach might be more appropriate if specific layers/cells are of interest, while the second approach appears to be more appropriate to give a representative score for the entire organ, since this is made up of the different components/structures (18). In this case, the accuracy of the assessment is increased if the significance, or “weight,” of each contributing index is determined, before any sum or average becomes meaningful.

### Should These Indices Be Measured at the End of the Trial or at Different Time Points to Provide a Meaningful Picture?

If we use soybean-induced enteritis in salmon, as an example, early alterations include: shortening of mucosal folds, reduced vacuolation of enterocytes and increased infiltration by inflammatory cells of the epithelium, lamina propria and maybe submucosa. Depending on the inclusion levels, these can even appear in a few days, followed by changes in the populations and morphology of goblet cells, as Urán et al. (9) and Sahlmann et al. (19) demonstrated. It should be noted that in the two publications mentioned, 4–5 fish per treatment were sampled per time point, so a higher sample size could have indicated early signs even earlier. For the case of goblet cells in particular, it should also be noted that chronic influence of various stressors may result in the loss of goblet cells (20) and thus their assessment might not be that valid if only performed after a prolonged treatment. Hence, when the development of any histological alteration is not known, multiple sampling points should be used to indicate the progress of the condition and support the conclusions.

## Author Contributions

IV: preparation of the entire manuscript.

## Conflict of Interest

The author declares that the research was conducted in the absence of any commercial or financial relationships that could be construed as a potential conflict of interest.
